# Protein Biomarkers in Venous Leg Ulcer Fluid: A Systematic Review

**DOI:** 10.1111/iwj.70675

**Published:** 2025-05-08

**Authors:** Renitha Reddi, Matthew Tan, Alun Huw Davies, Sarah Onida

**Affiliations:** ^1^ Section of Vascular Surgery, Department of Surgery & Cancer Imperial College London, Charing Cross Hospital London UK; ^2^ Royal College of Surgeons Ireland Dublin Ireland

**Keywords:** biomarkers, exudate, leg ulcer

## Abstract

Venous leg ulcers (VLUs) are common and cause significant morbidity and poor quality of life. There is a poor understanding of the biology underlying non‐healing VLUs. VLU exudates may reflect the underlying wound microenvironment. This systematic review aims to identify potentially diagnostic and/or prognostic protein biomarkers within VLU fluid/exudates reported in the literature. A systematic review was reported according to PRISMA guidelines. MEDLINE and Embase databases were searched up to 31st March 2024. Full text, primary studies in English reporting on proteins identified in VLU fluid/exudate were included. Two independent reviewers performed the abstract and full‐text screen. Additional publications were identified by searching the references of included studies. 46 studies were identified, with nine comparing healing and non‐healing VLUs. Cytokines (e.g., IL‐1a, IL‐1ra, IL‐6, eotaxin, GM‐CSF, PDGF, VEGF) and proteins involved in extracellular matrix (ECM) homeostasis (e.g., MMP‐7, MMP‐10, MMP‐13, TIMP‐4) were significantly increased in non‐healing compared to healing VLUs. Collagen subunits (PICP and PIIINP) significantly increased as the VLU healed. Inflammatory proteins (e.g., complement type 6, S100A8, S100A9) and ECM proteins (e.g., fibronectin, lumican) were found to be increased in non‐healing VLUs compared to acute surgical wounds. Altered levels of specific proteins in wound exudates may be indicative of healing and non‐healing VLUs. Further work is essential to elucidate a comprehensive protein phenotype that may help early identification and prognostication of non‐healing VLUs.

**Summary**

- Protein biomarkers that may be diagnostic and prognostic in chronic venous leg ulcer (VLU) fluid that have been identified previously were compiled in this study.
- The work presents an analysis of the proteins linked to healing and non‐healing in chronic venous leg ulcers.
- It highlights some of the important biomarkers that could guide the development of future therapeutic tools for focused therapy.
- The significance of precision biomarker‐based techniques is highlighted by this work, as they have the potential to enhance customised treatment plans, accelerate wound healing, and lower recurrence rates in patients with VLUs.

**Abbreviations**
AHSG: Alpha‐2‐HS‐glycoprotein
ANXA1: Annexin A1
bFGF: Basic Fibroblast Growth Factor
C6: Complement Component 6
CRP: C‐reactive Protein
ECM: Extracellular Matrix
ELISA: Enzyme‐Linked Immunosorbent Assay
Eotaxin: Eotaxin (CCL11)
Flii Protein: Flightless‐1 Protein
FLT‐3 L: FMS‐like Tyrosine Kinase 3 Ligand
Fractalkine: CX3CL1 (Fractalkine)
GM‐CSF: Granulocyte‐Macrophage Colony‐Stimulating Factor
HNE: Human Neutrophil Elastase
ICAM‐1: Intercellular Adhesion Molecule 1
IFN‐γ: Interferon Gamma
IL‐10: Interleukin‐10
IL‐12: Interleukin‐12
IL‐13: Interleukin‐13
IL‐15: Interleukin‐15
IL‐16: Interleukin‐16
IL‐17: Interleukin‐17
IL‐17a: Interleukin‐17A
IL‐1ra: Interleukin‐1 Receptor Antagonist
IL‐1α (IL‐1a): Interleukin‐1 Alpha
IL‐1β (IL‐1b): Interleukin‐1 Beta
IL‐2: Interleukin‐2
IL‐22: Interleukin‐22
IL‐3: Interleukin‐3
IL‐4: Interleukin‐4
IL‐5: Interleukin‐5
IL‐6: Interleukin‐6
IL‐9: Interleukin‐9
Interleukin‐8: IL‐8 (CXCL8)
JAK/STAT: Janus Kinase/Signal Transducer and Activator of Transcription Pathway
JNK: c‐Jun N‐terminal Kinase
KGF: Keratinocyte Growth Factor
LCN2 (NGAL): Neutrophil Gelatinase‐Associated Lipocalin
LPS: Lipopolysaccharide
LTF: Lactotransferrin
MAPK: Mitogen‐Activated Protein Kinase
MCP‐1 (CCL2): Monocyte Chemoattractant Protein‐1
MIP‐1α (CCL3): Macrophage Inflammatory Protein‐1 Alpha
MMP‐1: Matrix Metalloproteinase‐1
MMP‐10: Matrix Metalloproteinase‐10
MMP‐12: Matrix Metalloproteinase‐12
MMP‐13: Matrix Metalloproteinase‐13
MMP‐2: Matrix Metalloproteinase‐2
MMP‐3: Matrix Metalloproteinase‐3
MMP‐7: Matrix Metalloproteinase‐7
MMP‐8: Matrix Metalloproteinase‐8
MMP‐9: Matrix Metalloproteinase‐9
MPO: Myeloperoxidase
NAP2: Neutrophil‐Activating Peptide 2
NF‐κB: Nuclear Factor Kappa B
NGF: Nerve Growth Factor
PDGF: Platelet‐Derived Growth Factor
PDGF‐AA: Platelet‐Derived Growth Factor AA
PDGF‐AB: Platelet‐Derived Growth Factor AB
PDGF‐BB: Platelet‐Derived Growth Factor BB
PI3K: Phosphoinositide 3‐Kinase
PICP: Procollagen Type I C‐Terminal Propeptide
PIIINP: Procollagen Type III N‐Terminal Propeptide
Plasmin: Plasminogen‐Derived Enzyme
Pro‐MMP‐2: Pro‐Matrix Metalloproteinase‐2
Pro‐MMP‐9: Pro‐Matrix Metalloproteinase‐9
RANTES (CCL5): Regulated on Activation, Normal T Cell Expressed and Secreted
S100A8: S100 Calcium‐Binding Protein A8
S100A9: S100 Calcium‐Binding Protein A9
sEng (Soluble Endoglin): Soluble Endoglin
Soluble Thy‐1: sThy‐1 (Soluble Thy‐1)
sPARI: Soluble Plasminogen Activator Receptor Inhibitor
sTNFR‐II: Soluble TumourTumor Necrosis Factor Receptor II
suPARIII: Soluble Urokinase‐Type Plasminogen Activator Receptor III
TGF‐β: Transforming Growth Factor Beta
TGF‐β1: Transforming Growth Factor Beta 1
TGF‐β2: Transforming Growth Factor Beta 2
TGF‐β3: Transforming Growth Factor Beta 3
TIMP‐1: Tissue Inhibitor of Metalloproteinases‐1
TIMP‐2: Tissue Inhibitor of Metalloproteinases‐2
TIMP‐3: Tissue Inhibitor of Metalloproteinases‐3
TIMP‐4: Tissue Inhibitor of Metalloproteinases‐4
TNFr2: TumourTumor Necrosis Factor Receptor 2
TNF‐α: TumourTumor Necrosis Factor Alpha
TNF‐β: TumourTumor Necrosis Factor Beta
uPA: Urokinase‐Type Plasminogen Activator
uPA Antigen: Urokinase‐Type Plasminogen Activator Antigen
VEGF: Vascular Endothelial Growth Factor
VEGF‐165: Vascular Endothelial Growth Factor 165 Isoform
VEGF‐R1: Vascular Endothelial Growth Factor Receptor 1
VLU: Venous Leg Ulcer
vWF: Von Willebrand Factor
α1‐antitrypsin (AAT): Alpha‐1 antitrypsin
α1‐microglobulin (A1M): Alpha‐1 microglobulin

## 1 Introduction

Venous leg ulcers (VLUs) are a leading cause of morbidity and result in a significant reduction in health‐related quality of life for patients. It has been estimated that up to 1% of the adult population worldwide is affected, increasing to up to 4% in the elderly population [1].

VLUs are the result of a chain of intricate cellular and humeral events that are triggered by venous hypertension, often associated with venous reflux or obstruction and amplified by genetic predisposition [2]. Acute wounds normally go through time‐limited, consecutive stages (haemostasis, inflammation, granulation, and remodelling) that aid the restoration of anatomy and skin function. On the other hand, chronic VLUs are typically arrested in the inflammatory phase, which prevents progression to the following phases and delays wound closure [3].

Treatment for VLUs typically involves compression therapy, wound care, and superficial venous ablation [4]. Despite these interventions, up to 32% of patients do not experience complete healing, highlighting the need for further understanding and improvement in treatment strategies [4]. Compression therapy, which involves applying external pressure to the lower limbs to improve venous function, is the mainstay of treatment for established venous insufficiency [4]. Overall VLU healing rates have consistently averaged at 60%–70% after 6 months even with the gold standard of care, with a 50%–70% recurrence rate, suggesting that a sizable proportion of patients do not recover despite treatment efforts [5].

VLUs have several potential origin theories, including the formation of fibrin cuffs, leukocyte entrapment, and microangiopathy [1]. However, increased hydrostatic pressure‐induced inflammation in the venous circulation, which also results in increased ambulatory venous pressure, is the primary pathogenic mechanism in VLU. Leukocytes, notably macrophages and monocytes, T‐lymphocytes, inflammatory modulators and chemokines, cytokine production, growth factors, metalloproteinase activity, and other regulatory pathways are all involved in the inflammatory response [5]. Despite extensive research, the exact pathophysiological mechanisms underlying VLUs remain elusive, emphasising the need for continued investigation into the molecular processes involved [1].

Treatment inhibiting biomarkers in VLUs has been performed before with some success. A small pilot study from 2015 explored the use of adalimumab in 4 patients with refractory VLUs over a 4‐week period. They found that if the wound size decreased by over 25% in the 4 weeks, there was an associated decrease in wound tissue necrosis factor‐alpha (TNF‐α) levels [6], highlighting the translational potential of wound biomarker research in this field.

The wound fluid around an ulcer is thought to represent the wound microenvironment and can be collected non‐invasively [7]. Blood/plasma could be used to identify potential biomarkers; however, due to the invasive nature of venepuncture, it might not be suitable for routine monitoring. However, assessing systemic biomarker levels might not be an accurate method to assess the localised inflammation that occurs at the wound site specifically [8] Urine is a non‐invasive sampling method that could be used to sample biomarkers. Likewise, it can detect proinflammatory cytokines but can be influenced by systemic conditions [9].

This paper aims to explore the molecular aspects of VLUs, focusing on identifying potential biomarkers within wound fluid/exudates and understanding their implications for VLU diagnosis, treatment monitoring, and personalised care.

## 2 Materials and Methods

A systematic review of the literature was carried out according to the Preferred Reporting Items for Systematic Reviews and Meta‐Analyses (PRISMA) guidelines. This systematic review protocol was prospectively submitted to PROSPERO (reference no.: 328281).

The search was performed on 31st March 2024 from the MEDLINE and EMBASE databases for papers from 1947 up to and including that date by two reviewers using specific search terms. MeSH headings and other keywords pertaining to the target population were developed. This included terms relevant to patients with VLUs and protein biomarker assessment of venous ulcer fluid through any experimental methods. The full search strategy is as follows: (((chronic venous ulce*) OR (venous leg ulce*)) OR (venous ulce*)) AND ((((((ulcer fluid) OR (wound fluid)) OR (fluid)) OR (ulcer exudate)) OR (wound exudate)) OR (exudate)) AND ((protein) OR (proteomics) OR (biomarke*) OR (marke*)). Search results were uploaded to the online repository Covidence. Duplicates were identified and removed automatically. The remaining unique studies were independently screened using abstract review by two reviewers (MT and RR). Studies included after the abstract screen underwent a full‐text review to determine complete compliance with the inclusion and exclusion criteria. Any discrepancies during the screening process were discussed by the two reviewers and any disagreements were referred to a senior reviewer (SO). Finally, a thorough search of the references of selected publications was conducted to confirm that all relevant studies are included in this review.

Two reviewers worked independently to extract the data (RR and MT). Meta‐analysis was not possible due to the variability of the collection techniques used in studies and reporting of concentrations, and therefore only qualitative analysis was carried out. Where reported, information on protein concentrations was recorded.

### 2.1 Inclusion and Exclusion Criteria

Full text primary studies (randomised controlled trials, prospective cohort, retrospective cohort) in English reporting on protein biomarkers in VLU fluid/exudate in adults were included.

Studies reporting on metabolomic or non‐protein biomarkers only, biomarkers not identified from venous leg ulcer fluid (e.g., circulating biomarkers, arterial ulcers, diabetic ulcers, mixed ulcers), articles not reporting original research, articles not reported in English, conference abstracts, paediatric studies and animal studies were excluded.

### 2.2 Quality Assessment

Risk of bias assessment was performed using the Robins‐I tool for non‐randomised studies. The Newcastle‐Ottawa scale was used for cross‐sectional studies, and the modified Jadad scale was used for randomised controlled trials.

## 3 Results

### 3.1 Literature Search

**FIGURE 1:**
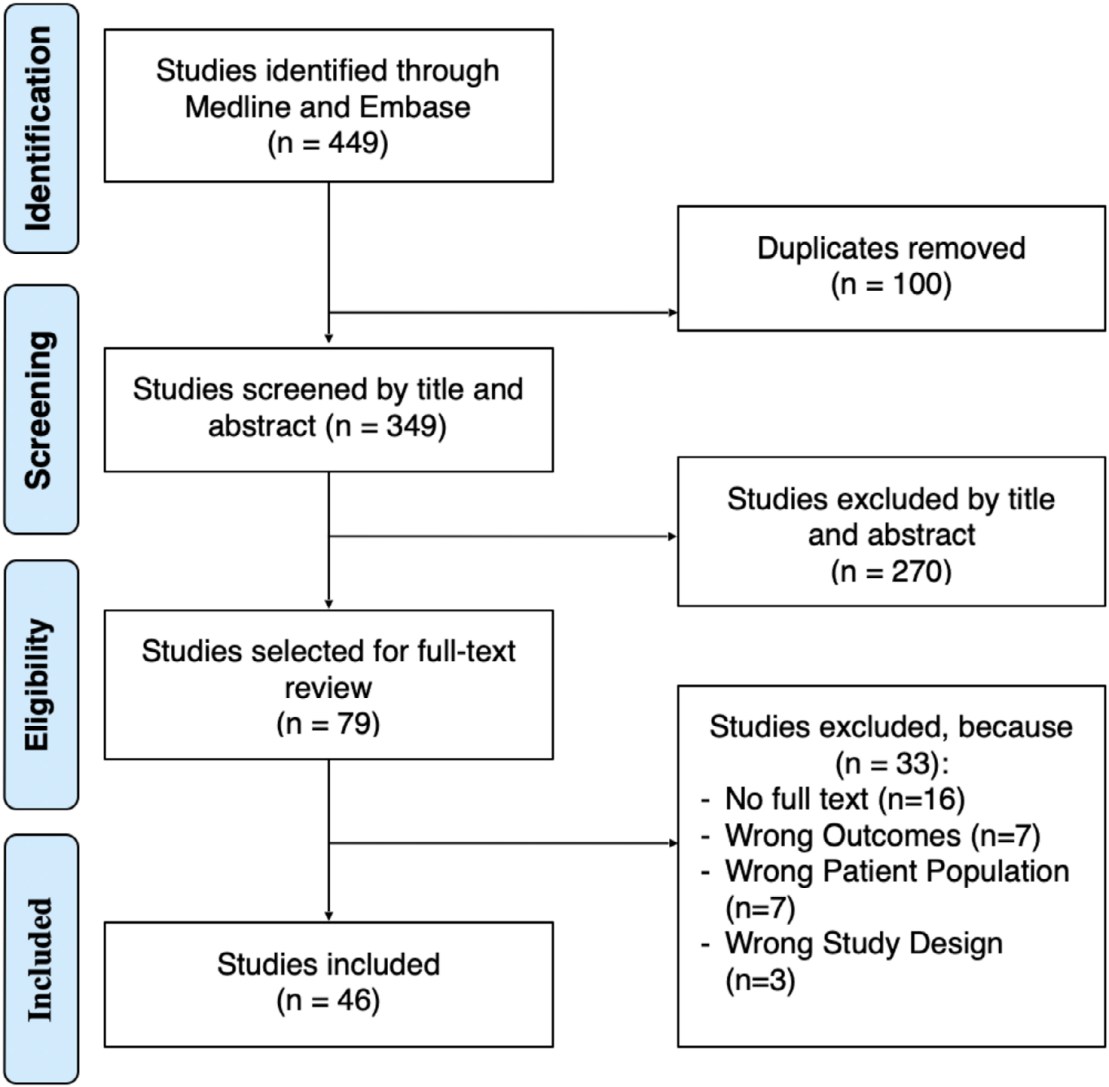
PRISMA diagram.

A total of 449 articles were identified using the search algorithm. 46 studies were included after screening (Figure 1). Included studies (Tables 1 and 2) were published from 1997 to 2023. Study designs consisted of six randomised controlled trials, 13 case–control studies, 15 cohort studies, seven cross‐sectional studies, and five interrupted time series studies. Enzyme‐linked immunosorbent assay (ELISA) was the most common analytical method of choice with additional targeted assays for specific markers. Other analytical methods are detailed in Tables 1 and 2.

**TABLE 1:** Healing versus non‐healing.

Author, year, country	Study design	Participant, sample size	Mean age, M:F	Comparator, sample size	Mean age, M:F	Ulcer aetiology	Analysis method	Comparison made	Upregulated proteins in healing	Downregulated proteins in healing	Proteins not significantly different
Ahmad, 2015, UK [10]	Cohort	9	62, 4:5	21	70, 10:11	Venous, DVT included	Time‐resolved fluorescence immunoassays Bioimmunoassay (uPA antigen) ELISA (PAI‐1 antigen)	Healing vs. non‐healing	sPARI, suPARIII		uPA antigen, PAI‐1 antigen
Amato, 2013, Italy [11]	Cohort	21	61.2, 8:13	24	68.7, 9:15	Venous	ELISA	Healing vs. non‐healing			MMP‐1, MMP‐8
Drinkwater, 2003, UK [12]	Case–control	19	NR	13	NR	Venous	ELISA	Healing vs. non‐healing		VEGF‐165	VEGF‐R1
Harris, 1995 [13]	Case–control	18	NR	NR	NR	Venous	ELISA, IL‐1 bioassay, IL‐6 bioassay	Healing vs. non‐healing			GM‐CSF, PDGB‐AB, BFGF, IL‐1a, IL‐1b, IL‐6
Kim, 2023 [14]	Cohort	74	71.8, 38:36	NR	NR	Venous	ELISA, fluorescence resonance energy transfer peptide	Healing vs. non‐healing		CRP, MMP	
Kirketerp‐Møller, 2022 [15]	Cohort	10	NR	7	NR	Venous	ELISA, Western Blot	Healing vs. non‐healing	MMP‐9		Protein, LPS, TNF‐α, HNE activity, MPO activity, MMP‐2, MMP‐8, TIMP‐1
McQuilling, 2022, USA [16]	Cohort	15	62.6, 0:15	NR	NR	Venous	Microarray	Healing vs. non‐healing	MMP‐7, MMP‐10, TIMP‐4	CX3CL1, FLT‐3 L, IL‐1a, IL‐1ra, IL‐2, IL‐3, IL‐9, MCP‐1, TNF‐β	
Mikosiński, 2022, Poland [17]	Cohort	57	69.2, 26:31	NR	NR	Venous	Mass spectrometry	Healing vs. non‐healing	TNF‐α	S100A8, S100A9, neutrophil elastase, MMP‐2	IL‐1b, MMP‐9, collagen a1, fibronectin
Mwaura, 2006, Ireland [18]	Cohort	40	60, 11:29	NR	NR	Venous, DVT excluded	ELISA	Healing vs. non‐healing	PDGF‐AA		TIMP‐2, MMP‐2
Pukstad, 2010, UK [19]	Interrupted time series	NR	NR	NR	NR	Venous	Antibody assay, ELISA	Healing vs. non‐healing	IL‐8, MIP‐1a	IL‐1a, IL‐1b, MIP‐1d, sTNFR‐II, Lipocalin‐2	
Stacey, 2019 [20]	Cohort	42	74.5, 1:1	NR	NR	Venous, DVT included	Multiplex ELISA	Healing vs. non‐healing	Albumin, Eotaxin, IL‐6, MCP‐1, PDGF‐BB, TIMP‐4	GM‐CSF, ICAM‐1, IL‐16, MIP‐1a, MMP‐13	

**TABLE 2:** Other studies.

Author, year, country	Study design	Participant, sample size	Mean age, %male	Comparator, sample size	Mean age, %male	Ulcer aetiology	Analysis method	Comparison made	Proteins identified	Upregulated proteins in chronic VLUs	Downregulated proteins in chronic VLUs
Allhorn, 2003, Sweden [21]	Cross‐sectional	12	NR	N/A	N/A	Venous	Gel electrophoresis, Immunoblotting	N/A	Hemea1‐microglobulin		
Cavassan, 2019, Brazil [22]	Cross‐sectional	28	70 ± 10.1, 5:23	N/A	N/A	Venous, post thrombotic included	LCMS	N/A	Untargeted		
Cavallo, 2022, Italy [23]	Case–control	45	81, 17:28	56	76, 24:32	Venous	NR	Infected vs. non‐infected	IFN‐g, IL‐1b, IL‐6, IL‐8, IL‐10, IL‐17a, IL‐22, TNF‐α		
Cowin, 2006, Australia [24]	Cross‐sectional	16	52–88, NR	NR	NR	Venous	ELISA	Acute surgical wound	TNF‐α	TNF‐α	
Da Silva, 2014, Brazil [25]	Cross‐sectional	6	NR	N/A	N/A	Venous	Zymography	N/A	MMP‐2, MMP‐9		
Eming, 2010 [26]	Case–control	19	67, NR	9	65, NR	Venous	LCMS, ELISA, Antibody assay	Acute surgical wound	Untargeted	Fibrinogen y/b‐chain, vitronectin, fibronectin, lumican, a2‐HS‐glycoprotein, C6, S100A8, S100A9, Lactotransferrin, ANXA1	
Fivenson, 1997 [27]	Interrupted time series	14	NR	N/A	N/A	Venous	ELISA	N/A	Untargeted		NAP2
Gohel, 2008, UK [28]	Cohort	80	75 (40–93), 43:37	N/A	N/A	Venous, 12 previous DVT	ELISA, Gelatin zymography	N/A	TNF‐α, IL‐1b, bFGF, VEGF, TGF‐Β1 proMMP‐2, proMMP‐9		TGF‐Β1
Goto, 2016, Japan [29]	Cross‐sectional	13	73 (47.5–76.5), 7:6	N/A	N/A	Venous	ELISA	N/A	NGF, S100A8, S100A9		
Grzela, 2014, Poland [30]	Cohort	8	68.3 ± 9.3, 3:5	N/A	N/A	Venous, all superficial venous insufficiency, DVTs excluded	Gelatin/SDS zymography	N/A	MMP‐2, MMP‐9		
Lauer, 2000 [31]	Case–control	15	67, NR	NR	NR	Venous, both primary and secondary	ELISA	Acute wound	VEGF, Plasmin	VEGF	
Ligi, 2017 [32]	Case–control	30	77.9 ± 6.6, NR	NR	71.4 ± 14.4, NR	Venous, DVTs not reported	Multiplex immunoassay	Granulating vs. Inflammatory	TGF‐β isoforms (B1, B2, B3), sEng	TGF‐Β3, sEng	
Ligi, 2016a [33]	Case–control	34	77.8 ± 6.5, NR	NR	69.1 ± 14.8, NR	Venous, deep and mixed reflux included	Multiplex immunoassay	Granulating vs. Inflammatory		MMP‐2, MMP‐9, MMP‐12, TIMP‐1, TIMP‐2	MMP‐1, MMP‐7, MMP‐13, TIMP‐4
Ligi, 2016b [34]	Case–control	34	77.8 ± 6.5, NR	NR	69.1 ± 14.8, NR	Venous, deep and mixed reflux included	Multiplex immunoassay	Granulating vs. Inflammatory		IL‐1B, IL‐12, IL‐15, IL‐17, IFN‐γ, TNF‐α, IL‐4, IL‐8/CXCL8, Eotaxin/CC 11, MCP‐1/CCL, GM‐CSF	IL‐6, IL‐5, RANTES/CCL5
Litwiniuk, 2012, Poland [35]	Cohort	25	76.3 ± 12, 9:16	N/A	N/A	Venous	Gelatin/SDS zymography	N/A	MMP‐2, MMP‐9		
McDaniel, 2017, USA [36]	RCT	35	60.3 ± 12.6, 5:3	NR	60.9 ± 11.8, 11:8	Venous	ELISA	EPA + DHA supplementation effect on wound healing vs. control	MMP‐8, Neutrophil elastase		
McInnes, 2014, Wales [37]	Case–control	20	41–91, 13:7	NR	NR	Venous	Microarray, Zymography, ELISA	Infected vs. non infected	Angiogenin, IL‐1b, IL‐2, IL‐4, IL‐5, IL‐6, IL‐8, IL‐10, IL‐12p40, IL‐12p70, IL‐13, TNF‐α, TNFr2, IFN‐γ, TGF‐β1, VEGF, ICAM‐1, IFN‐γ inducible protein 1, elastase, a1‐antitrypsin		
Mendez, 1999, USA [38]	Cohort	NR	NR	N/A	N/A	Venous	ELISA	N/A	TNF‐α		
Moor, 2009, USA [39]	Cohort	4	60.5, NR	N/A	N/A	Venous	Bioassay, ELISA, Zymography, Multiplex immunoassay	N/A	MMP‐13, MMP‐1, MMP‐9, HNE, MMP‐8, MMP‐2		
Paul, 2021, USA [40]	Cohort	14	62.14 ± 4.52, 9:5	N/A	N/A	Venous, all IVDUs	ELISA	N/A	CRP		
Rasmussen, 1992, Denamark [41]	Interrupted time series	14	70, 5:9	N/A	N/A	Venous	Radioimmunoassay	N/A	Collagen type III propeptide, Collage type I propeptide		
Ruzehaji, 2012, Australia [42]	Cross‐sectional	6	80, 1:2	N/A	N/A	Venous	Western blotting	N/A	Flii protein		
Saalbach, 1999, Germany [43]	Case–control	8	NR	N/A	N/A	Venous	ELISA	N/A	sThy‐1		
Senet, 2003, France [44]	RCT	8	72.3 (45–88), 3:4	7	72.3 (50–83), 1:1	Venous, both primary and secondary, PTS 5 patients (3 intervention, 2 control)	ELISA	Effect of autologous platelets as adjuvant therapy for venous ulcer healing vs. control	IL‐8, VEGF, KGF, TIMP‐1		
Serra, 2013, Italy [44]	Interrupted time series	31	51.5 ± 9 (42–61), 9:22	20	52.3 ± 8.5 (45–58), 3:4	Venous, all superficial ± perforator insufficiency, no deep insufficiency but no DVT history reported	ELISA	Acute surgical wound	NGAL, MMP‐9	NGAL, MMP‐9	
Serra, 2015, Italy [45]	RCT	32	50.5 ± 8 (41–60), 9:23	32	51.3 ± 7.5 (46–57), 11:21	Venous, all superficial ± perforator insufficiency, no deep insufficiency but no DVT history reported	ELISA	Effect of doxycycline on ulcer healing vs. control	NGAL, MMP‐9, VEGF	NGAL, MMP‐9	
Siquiera, 2015, Brazil [46]	RCT	10 (6 unilateral, 4 bilateral)	58.03 ± 17.83, 67.5 ± 11.38, NR	5	54.8 ± 8.58	Venous	ELISA	Healing rate after LED irradiation vs. control	TNF‐α		
Smeets, 2008, Germany [47]	RCT	17	68 ± 9, NR	10	66 ± 10, NR	Venous	ELISA	ORC/collagen matrix vs. control	MMP‐2		
Smith, 2015, UK [48]	Cross‐sectional	6	45–86, 5:1	N/A	N/A	Venous	Western blotting	N/A	Angiostatin, Endostatin, Glypican‐1		
Tarlton, 1999, UK [49]	Cohort	26	NR	N/A	N/A	Venous	Gelatin zymography, Casein zymography, ELISA	N/A	Pro‐MMP‐2, Pro‐MMP‐9, MMP‐2, MMP‐9, Neutrophil elastase, Type I collagen propeptide		
Trøstrup, 2011, Denmark [50]	Case–control	9	NR	NR	NR	Venous	Microarray, Zymography, ELISA	Versus diabetic foot ulcer and acute wound	CRP fibronectin vWF S100A8/A9	CRP, fibronectin, vWF	S100A8/A9
Trøstup, 2018, Denmark [51]	Case–control	8	81 ± 7, 4:4	NR	NR	Venous	ELISA	Versus diabetic foot ulcer and acute wound	MMP‐9, TIMP‐1		MMP‐9
Wiegand, 2017 [52]	RCT	36	NR	NR	NR	Venous	Multiplex immunoassay	Effect of noncontact low‐frequency US on wound healing vs. control	IL‐1b, TNF‐α, IL‐6, IL‐8, IL‐10		
Wysocki, 1999 [52]	Case–control	11	63.6 (37–94), 8:3	NR	NR	Venous	Zymography, Western blotting	N/A	uPA, PAI‐1, MMP‐9		
Zillmer, 2011, Denmark [53]	Interrupted time series	23	Cohort 1: 80 ± 3 (50–87), 9:2 Cohort 2: 73 ± 3 (49–84), 1:2	N/A	N/A	Venous	ELISA	N/A	IL‐1α, IL‐1β, IL‐8, MMP‐9		

Eleven studies compared healing to non‐healing VLUs [7, 8, 9, 10, 11, 12, 13, 14, 15, 16, 17]. Six studies compared chronic VLUs to acute surgical and/or burn wounds [24, 26, 31, 45, 51, 54].

### 3.2 Quality Assessment Report

Overall risk of bias was moderate across all studies. This was due to varying inclusion and exclusion criteria for study participants, with few studies actively excluding a history of deep venous thrombosis (DVT) as well as different definitions for chronic VLU. The randomised controlled trials (RCTs) were all rated as high quality by the modified Jadad, with the six studies scoring between four and eight (Table A1). The Newcastle‐Ottawa scale rated three of the cross‐sectional studies as high quality and the remaining four as fair quality according to the Agency for Healthcare Research and Quality standards (Table A2). The overall bias for the remaining non‐randomised studies (Table 3) according to the Robins‐I scale was low–to–medium, with only three studies having severe bias.

**TABLE 3:** Robins‐I scale for non‐randomised studies.

Study	Bias due to confounding	Bias in selection of participants into the study	Bias in classification of interventions	Bias due to deviations from intended interventions	Bias due to missing data	Bias in measurement of outcomes	Bias in selection of the reported result	Overall bias
Ahmad, 2015								
Amato, 2013								
Cavallo, 2022								
Drinkwater, 2003								
Harris, 1995								
McQuilling, 2022								
Mwaura, 2006								
Pukstad, 2010								
Stacey, 2019								
Trostrup, 2018								
Ewing, 2010								
Fivenson, 1997								
Gohel, 2008								
Grzela, 2014								
Kim, 2023								
Kirketerp‐Møller, 2022								
Lauer, 2000								
Ligi, 2017								
Ligi, 2016								
Ligi, 2016 (2)								
Litwinuik, 2012								
McInnes, 2014								
Mendez, 1999								
Mikosiński, 2022								
Moor, 2009								
Paul, 2021								
Rasmussen, 1992								
Saalbach, 1999								
Serra, 2013								
Tarlton, 1999								
Trøstrup, 2018								
Trøstrup, 2011								
Wysocki, 1999								
Zillmer, 2011								

*Note:* Green: low bias; Orange: moderate bias; Red: severe bias.

### 3.3 Healing Versus Non‐Healing

Twelve of the 46 included studies compared protein levels found in the ulcer fluid of healing and non‐healing chronic VLUs. The results of the most commonly reported biomarkers in the studies that discussed healing and non‐healing VLUs are reported in Table 4.

**TABLE 4:** Biomarkers assessed in multiple studies‐healing vs. non‐healing VLUs.

TIMP‐4	⇑ [16]	⇑ [20]	
MCP‐1	⇑ [20]	⇓ [16]	
MIP‐1a	⇑ [19]	⇓ [20]	
GM‐CSF	⇔ [13]	⇓ [20]	
Il‐1a	⇔ [13]	⇓ [16]	⇓ [19]
Il‐1b	⇔ [13]	⇔ [17]	⇓ [19]

*Note:* ⇑ increased in healing VLUs compared to non‐healing VLUs; ⇔ no change; ⇓ decreased in healing VLUs compared to non‐healing VLUs.

When considering the levels of cytokines in healing versus non‐healing VLUs, an early small case–control study found that cytokines involved in acute inflammation (e.g., (interleukin‐1 alpha (IL‐1a), interleukin‐1 beta (IL‐1b)) and in the switch from an acute to chronic inflammatory state (e.g., interleukin‐6 (IL‐6)) had similar levels in fluid obtained from both healing and non‐healing VLUs [12]. However, a more recent cohort study refutes this finding. In addition to the IL‐1 cytokine superfamily, other inflammatory cytokines interleukin‐2 (IL‐2), interleukin‐3 (IL‐3), interleukin‐9 (IL‐9), and tissue necrosis factor beta (TNF‐β) were found to be downregulated in the fluid from healing VLUs [16]. In a larger cohort study, IL‐6 was also shown to be elevated in healing VLUs when compared to non‐healing VLUs, consistent with its role as a local modulator of inflammation and regulator of skin repair [20].

While the specific biochemical factors that contribute to healing in VLUs are unclear, several metalloproteinases appear to be involved in the initiation and healing phases [11]. A cohort study comparing healing and non‐healing VLUs found decreased levels of matrix metalloproteinase‐13 (MMP‐13) in healing VLUs, which is involved in extracellular matrix degradation [20]. Another cohort study reported elevated levels of matrix metalloproteinase‐7 (MMP‐7) and matrix metalloproteinase‐10 (MMP‐10), both of which are involved in wound healing in healing compared to non‐healing VLUs [16]. Tissue inhibitors of metalloproteinase‐4 (TIMP‐4) is a key regulator of matrix metalloproteinase‐9 (MMP‐9) and also has a role in controlling the recruitment and aggregation of platelets [55]. Both cohort studies found elevated levels of TIMP‐4 in healing VLUs compared to non‐healing VLUs [16, 20]. MMP‐9 was shown to elevated compared to non‐healing VLUs in a different cohort study [15].

Chemokines, growth factors and colony stimulating factors also appear to play a role in VLU chronicity. A cohort study reported elevated levels of monocyte chemoattractant protein‐1 (MCP‐1) in healing compared to non‐healing VLUs [20]. These results were not replicated in a second cohort study, where decreased levels of MCP‐1 were seen in healing VLUs [16]. Another monocyte chemoattractant, macrophage inflammatory protein‐1 alpha (MIP‐1a) was shown to be increased in healing chronic VLUs compared to non‐healing VLUs [19]. However, the opposite was reported in a study conducted in 2019 [20]. Levels of another essential growth factor for monocyte function, granulocyte‐macrophage colony‐stimulating factor (GM‐CSF), were shown to be similar between healing and non‐healing VLUs in an early case control study [13]. Conversely, a more recent study reported decreased levels of GM‐CSF in healing VLUs compared to non‐healing [20]. This is unexpected as a suggested function of GM‐CSF is promoting wound healing [56].

### 3.4 Other Studies

Some studies compared the levels of protein biomarkers in chronic VLU and acute surgical wound fluid (Table 5). Increased levels of vascular endothelial growth factor (VEGF) were seen in an initial case–control study in chronic VLU fluid [31]. A cross‐sectional study in 2006 found increased levels of the cytokine TNF‐α in VLU compared to acute surgical wound fluid [24]. TNF‐α, produced by macrophages, stimulates fibroblasts to produce proteoglycan and fibronectin, which helps the extracellular matrix develop in damaged tissues [7]. The levels of extracellular matrix (ECM) and inflammatory proteins were also seen to be altered in chronic venous ulcer fluid compared to acute surgical wound fluid. A case control study found significantly elevated levels of fibronectin, S100 calcium‐binding protein A8 (S100A8) and S100 calcium‐binding protein A9 (S100A9) among others [26]. In a different study, fibronectin was shown to be elevated, but S100A8 and S100A9 were decreased in chronic venous ulcer fluids compared to acute wound fluid [51]. A previous interrupted time series found elevated levels of neutrophil gelatinase‐associated lipocalin (NGAL) and MMP‐9 in chronic venous ulcer fluid [45]. MMP‐9 is regulated by NGAL and is released by neutrophils [57].

**TABLE 5:** Biomarkers assessed in multiple studies—VLU vs. acute wounds.

Fibronectin	⇑ [26]	⇑ [51]
S100A8 and S100A9	⇑ [26]	⇓ [51]

Several studies compared chronic VLU fluid using normal plasma as a baseline (Table 6). Through the stimulation of proangiogenic cytokines like TNF‐α and VEGF and the production of antiangiogenic peptides, MMP‐2 and MMP‐9 both contribute to the regulation of angiogenesis required for wound healing [55]. There was no significant difference in the levels of MMP‐2 [25, 30, 35, 39, 50] or MMP‐9 [25, 30, 35, 39, 50, 53, 58] found in the studies that compared chronic venous ulcer fluid to plasma. There was no difference in the levels of the cytokine IL‐1b [28, 53] or TNF‐α [28, 39]. There was also no change seen in the levels of growth factors such as basic fibroblast growth factor (bFGF), VEGF, and nerve growth factor (NGF) in both fluids [28, 29]; however, one study showed decreased levels of tissue growth factor‐beta (TGF‐b) in chronic venous ulcer fluid [28]. TGF controls re‐epithelialization, as well as inflammation, angiogenesis, and the development of granulation tissue during the healing of wounds [59]. The low levels of TGF‐β in chronic venous ulcer fluid could explain the arrested healing.

**TABLE 6:** Biomarkers assessed in multiple studies—VLU vs. plasma levels.

MMP‐2	⇔ [25, 30, 35, 39, 50]
MMP‐9	⇔ [25, 30, 35, 39, 50, 53, 58]
IL‐1b	⇔ [28, 53]
TNF‐α	⇔ [28, 39]
bFGF, VEGF and NGF	⇔ [28, 29]

*Note:* ⇑ increased in VLUs; ⇔ no change; ⇓ decreased in VLUs.

## 4 Discussion

This systematic review has identified several different studies that investigated the same biomarkers and reported conflicting results. These differences may be due to the methodological differences in the studies, including the definition of patient groups. For example, IL‐6 was shown to be upregulated in one study on healing versus non‐healing chronic VLUs [20] but downregulated in another which compared “inflammatory” and “granulating” VLUs [34]. While a similar number of patients and age ranges were seen in both studies, the study that showed elevated levels of IL‐6 included patients with DVTs and used multiplex ELISA for detection rather than a multiplex immunoassay used in the second study. Another protein that had variation between studies was TGF‐β. It was shown to be decreased in healing chronic venous wounds, which included patients with DVTs, in an earlier study [28], but increased in a more recent study which did not include DVTs [32]. Again, there was a difference in the inclusion of patients with DVTs and protein identification methods. There was also variation in the levels of MIP‐1a reported. It was reported to be upregulated in healing chronic VLUs [19] but downregulated in a later study [20]. Both studies used the same identification method, ELISA, and evaluated the changing levels of the biomarker over time but details on patient characteristics were not available in the first study. The former study used an occlusive dressing to collect the wound fluid, whereas the latter used needle aspiration. MCP‐1 was another protein which had different levels in 2 cohort studies. One study found elevated levels of MCP‐1 and used ELISA for detection and needle aspiration to collect the fluid [20]. The study that found decreased levels of MCP‐1 used proteomic microarrays for detection and used a swab to collect the exudate [16].

The heterogeneity in the papers included can be due to several reasons. Firstly, variations in study design, including differences in sample size, participant demographics, and inclusion criteria, can contribute to this. For instance, variations in VLU aetiology, such as the inclusion or exclusion of DVTs and post‐thrombotic syndrome, can influence the underlying pathophysiology and thus the biochemical profile of VLUs. As well, different methods of protein analysis can yield differing results. A targeted search compared to an untargeted analysis for proteins may lead to varying outcomes. Additionally, the inherent complexity of chronic wounds, influenced by factors such as co‐morbidities, concomitant medication use, and wound chronicity, introduces variability across studies.

The diversity within patient populations across various studies can significantly influence the outcomes and interpretations of the findings presented here. Factors such as age, gender distribution, comorbidities, ulcer aetiology, and geographical location can introduce variations in the patterns of protein expression and wound healing. For instance, differences in mean age and gender ratios among participants may contribute to variations in the inflammatory response and tissue repair mechanisms. Geographical and regional disparities may impact genetic predispositions, environmental influences, and healthcare access, further influencing the observed protein translation patterns and treatment responses. Considering these diverse patient populations in future omic studies will be crucial for contextualising and generalising research findings to broader clinical settings and populations.

The included studies have varying methods, testing time points, methods of data collection and analysis. The most common wound fluid collection methods were using cotton buds/gauze to collect the fluid and occlusive dressing followed by needle aspiration, with the more popular method being the latter. There is no gold standard for wound fluid collection, but methods can depend on the size and nature of the wound [60]. There were variations in the time frames used to define a wound as healing or non‐healing. The length of time reported for a venous ulcer to be considered non‐healing in studies that directly compared these patient populations ranged from 2 months to 1 year. The most common cut‐off point used to differentiate the groups was 2 months [10, 11, 12, 13, 14, 15, 16, 17, 18, 19, 20]. In the literature, there was no consensus as to a duration that defines a chronic, non‐healing venous ulcer.

Studies have been searching for biomarkers in isolation using different methods like ELISA, multiplex immunoassays, western blotting, zymography and biomarker specific assays. Currently, there is a lack of standardisation across the literature. However, the most common method used to detect proteins in VLU fluid in this study was ELISA, which, while it is the current gold standard [59], is increasingly superseded by high‐throughput analytical methods such as liquid chromatography– mass spectrometry.

**FIGURE 2:**
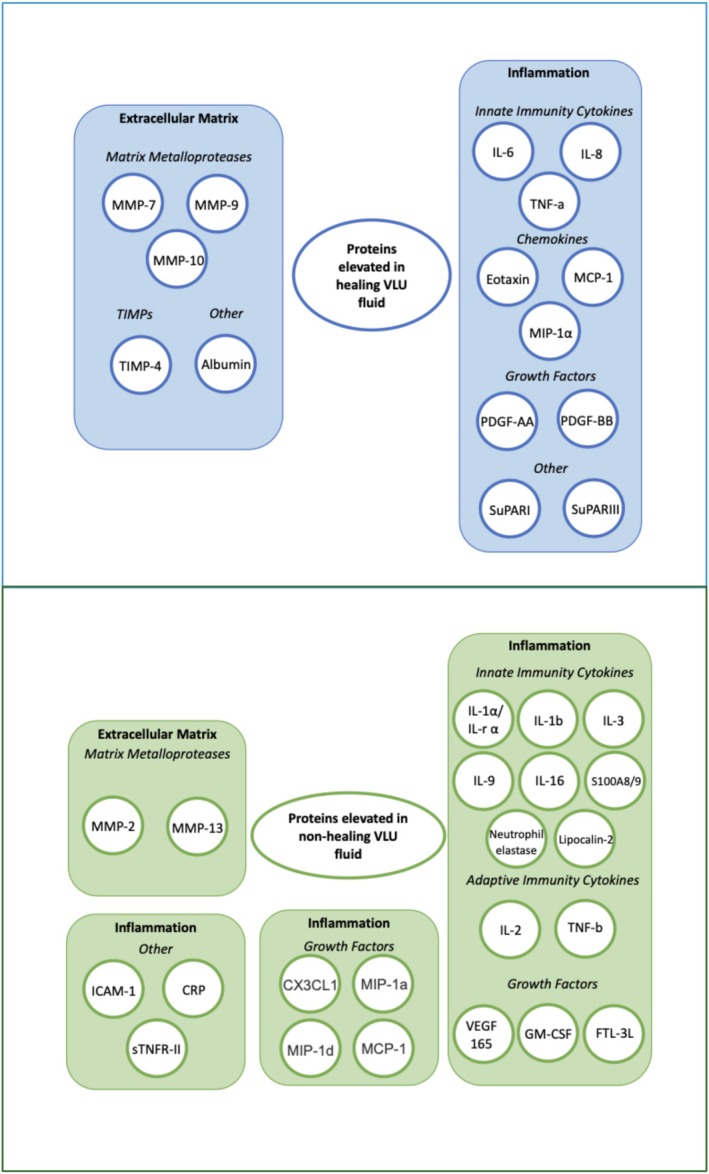
Schematic of proteins elevated in healing and non‐healing chronic venous ulcers.

Figure 2 highlights the proteins that have been found in this systematic review to be differentially expressed in healing and non‐healing ulcers. However, there is a lack of agreement on what makes a suitable biomarker. In this review, a number of proteins were identified that may represent important biomarkers in VLU healing. TIMP‐4 was the protein most frequently found to be increased in healing VLUs. This protein is a key modulator of MMP‐9, which was another protein occasionally increased in chronic VLUs. Though their affinity levels may vary, TIMPs can inhibit all MMPs by attaching to the active or alternative sites of MMPs. MMPs are crucial for wound healing; however, an imbalance in their levels may hinder the healing process [60]. TIMPs have a major function in controlling cell migration during wound healing by modifying MMP activity [61]. IL‐1a, the protein most frequently increased in non‐healing VLUs, is an agonist cytokine and is known to be involved in inflammation and wound healing [56].

Based on this systematic review and the data provided, the authors suggest that future studies on biomarkers in healing and non‐healing VLUs focus on the following specific biomarkers: TIMP‐4 and IL‐1a. TIMP‐4, a key regulator of MMP activity, was frequently increased in healing VLUs and may be a valuable therapeutic target. TIMP‐4 has not been extensively studied in the context of wound healing, but its potential role in vascular smooth muscle healing has been studied. TIMP‐4 is also thought to control gene expression, differentiation, migration, proliferation, apoptosis, and other cellular processes linked to the creation of neointima [62]. An in vivo study has suggested that TIMP‐4 overexpression significantly raised apoptotic cell death in vascular smooth muscle while having no effect on proliferation [62]. This could be one of the causes of increased TIMP‐4 in healing VLUs compared to non‐healing VLUs. Similarly, IL‐1a, which is elevated in non‐healing VLUs, plays a critical role in early inflammation and tissue repair but can hinder healing when persistently expressed. IL‐1a is released into the extracellular space upon cellular damage or programmed cell death, and it can alert neighbouring cells to impending damage or infection [63]. The process of wound healing benefits from early IL‐1a expression, which is necessary and increases the synthesis of collagen and the proliferation of keratinocytes and fibroblasts. After the first week of recovery, however, elevated levels of IL‐1a can be harmful and prevent wound healing [64]. A lower level in healing ulcers would be consistent with the dampening of the acute inflammatory response in favour of the regulatory inflammatory response required for wound healing.

With the caveat of the heterogeneity of the included studies as described above, the prevalence of these two proteins and the consistency of their expression levels related to VLU healing status across multiple studies suggest that they may represent key mediators in promoting and impairing VLU healing. Future wound studies should not only focus on determining the variation of their levels in VLUs but examine the downstream proteins and metabolites that are modulated by these proteins. When IL‐1a binds to its receptor complex (IL‐1 receptor (IL‐1RI) and IL‐1 receptor accessory protein (IL‐1RAcP)), it triggers a cascade that activates mitogen‐activated protein kinases (MAPKs), including extracellular signal‐regulated kinase (ERK), jun N‐terminal kinase (JNK), and p38. These kinases regulate various cellular processes by phosphorylating transcription factors, influencing gene expression related to inflammation, cell proliferation, and apoptosis [65]. Beyond MMP regulation, in the context of cancer, TIMP‐4 can influence various signalling pathways, including Notch, Hedgehog, TGF‐β, phosphatidylinositol 3‐kinase (PI‐3K), nuclear factor kappa B (NFκB), and Janus kinase (JAK) signal transducer and activator of transcription (JAK/STAT), which are essential for tumour progression and maintenance [66]. For TIMP‐4, its expression can be induced by TGF‐β1, and this induction is mediated partly by the ERK pathway and Sp1 transcription factor [67]. TIMP‐4 primarily inhibits MMP‐2 and MMP‐7, and to a lesser extent, MMP‐1, MMP‐3, and MMP‐9, suggesting its role in maintaining ECM integrity during tissue repair [68]. TIMP‐4 shares some functions with TIMP‐3, particularly in vascular regulation, but it does not seem to play as dominant a role as TIMP‐1 in direct ECM protection during epithelial migration. These findings suggest that targeting TIMP‐4 in chronic wounds might be beneficial in controlling angiogenesis and stabilising vasculature, rather than directly promoting keratinocyte migration [69]. Examining downstream processes of these proteins in the context of VLU patients would allow for more comprehensive mapping of pathways related to VLU pathogenesis and persistence and potentially provide information on up‐ and downstream proteins that may be stimulated or inhibited to promote VLU healing.

In summary, this systemic review has explored biomarkers present in venous leg ulcer fluid in relation to healing and non‐healing VLU, identifying specific proteins with diagnostic/prognostic value. To advance this field, we propose the following: (1) developing standardised definitions and methodologies for biomarker analysis; (2) standardisation of wound fluid sample collection; (3) employing advanced techniques such as liquid chromatography mass spectrometry to comprehensively examine the samples; and (4) prioritising targeted studies on TIMP‐4 and IL‐1a. This will ensure that findings are reproducible and suitable for comparison between studies.

## Conflicts of Interest

The authors declare no conflicts of interest.

## Data Availability

The data that support the findings of this study are available from the corresponding author upon reasonable request.

## Appendix A

**TABLE A1:** Modified Jadad Scale for bias in RCTs.

Corresponding author	Was the research described as randomized?	Was the approach of randomization appropriate?	Was the research described as blinding?	Was the approach of blinding appropriate?	Was there a presentation of withdrawals and dropouts?	Was there a presentation of the inclusion/exclusion criteria?	Was the approach used to assess adverse effects described?	Was the approach of statistical analysis described?	Total
McDaniel, 2017	y	y	y	y	y	y	y	y	8
Senet, 2003	y	n/a	y	n/a	y	y	y	y	6
Serra, 2015	y	n/a	y	n/a	n	y	n	y	4
Siquiera, 2015	y	n/a	y	y	y	y	n	y	6
Smeets, 2008	y	n/a	y	n/a	n	y	y	y	4
Wiegand, 2017	y	n/a	n	n/a	y	y	y	y	5

Abbreviations: y, yes; n, no; n/a, not applicable.

**TABLE A2:** Newcastle‐Ottawa Scale for the risk of bias and quality assessment of cross sectional studies.

Author	Year	Selection				Comparability	Exposure			Total score
		Adequate definition of patient cases	Representativeness of patient cases	Selection of controls	Definition of controls	Control for important or additional factors	Ascertainment of exposure	Same method of ascertainment for participants	Nonresponse rate	
Allhorn	2003	★	★		★	★	★	★		6
Cavassan	2019	★	★			★★	★	★		6
Cowin	2006	★	★			★★	★	★		6
Da Silva	2014	★	★			★	★	★		5
Goto	2016	★	★			★	★	★		5
Ruzehaji	2012	★	★		★	★★	★	★		7
Smith	2015	★	★			★★	★	★		6
Trøstrup	2011	★	★		★	★	★	★		6

* One point.
